# *Listeria monocytogenes* Meningitis in an Immunosuppressed Patient with Autoimmune Hepatitis and IgG4 Subclass Deficiency

**DOI:** 10.1155/2015/102451

**Published:** 2015-10-19

**Authors:** Shahin Gaini

**Affiliations:** ^1^Infectious Diseases Division, Medical Department, National Hospital Faroe Islands, 100 Tórshavn, Faroe Islands; ^2^Infectious Diseases Research Unit, Odense University Hospital and University of Southern Denmark, 5000 Odense, Denmark

## Abstract

A 51-year-old Caucasian woman with *Listeria monocytogenes* meningitis was treated and discharged after an uncomplicated course. Her medical history included immunosuppressive treatment with prednisolone and azathioprine for autoimmune hepatitis. A diagnostic work-up after the meningitis episode revealed that she had low levels of the IgG4 subclass. To our knowledge, this is the first case report describing a possible association between autoimmune hepatitis and the occurrence of *Listeria monocytogenes* meningitis, describing a possible association between *Listeria monocytogenes* meningitis and deficiency of the IgG4 subclass and finally describing a possible association between *Listeria monocytogenes* meningitis and immunosuppressive therapy with prednisolone and azathioprine.

## 1. Introduction

*Listeria monocytogenes* is a pathogen associated with meningitis and/or sepsis [1, 2]. It is well known that cases of invasive infections with* Listeria monocytogenes* are often associated with different states of immunosuppression and pregnancy, and with severe comorbidity like cancer [3–6]. Our case report is pointing towards three possible associations with invasive listeria infection: autoimmune hepatitis, treatment with prednisolone and azathioprine, and finally low levels of IgG4 subclass immunoglobulins.

## 2. Case Presentation

A 51-year-old Caucasian female, treated in the Hepatology Out-Patient Clinic with oral prednisolone 5 mg daily and oral azathioprine 100 mg daily for an autoimmune hepatitis since 2006, was admitted to the Medical Department at the National Hospital, Faroe Islands, in 2013, after only 12 hours with evolving headache, vomiting, and fever. At admission, the Glasgow Coma Scale (GCS) was 15 points, temperature 38.9 degrees Celsius, blood pressure 126/73, heart rate 85, and oxygen saturation 93%. No neck stiffness and no neurological deficits were found. Blood chemistry at the time of admission showed an elevated C-reactive protein (CRP) of only 13 mg/L. A CT of the brain without contrast and a chest X-ray were performed with no abnormalities. Blood cultures were drawn before administration of antibiotics. Because the patient had been coughing for 3 weeks prior to the admission, intravenous benzylpenicillin 2 MIU four times daily was started at the time of admission, on suspicion of lower respiratory tract infection. Within a few hours after admission, the patient became somnolent with descending GCS and rising fever to over 40 degrees Celsius. A spinal tap was performed and a turbid cerebrospinal fluid (CSF) was sampled with pleocytosis 1333 × 10^6^/L, 78% neutrophils, CSF-protein 2.0 g/L, and CSF-glucose 1.9 mmol/L (blood glucose 6.0 mmol/L). On suspicion of meningitis and/or encephalitis, the patient was switched over to intravenous benzylpenicillin 3 MIU 6 times daily combined with intravenous ceftriaxone 4 g daily and also combined with intravenous aciclovir 750 mg three times daily. The patient was not given dexamethasone, because she had received one dose of intravenous benzylpenicillin 2 MIU prior to the spinal tap. Gram stain of the CSF was negative. CSF was sent to polymerase chain reaction (PCR) examinations for Herpes simplex virus, Varicella-zoster virus, Epstein-Barr virus, Cytomegalovirus,* Mycoplasma pneumoniae*, and Enterovirus, specific PCR for* Streptococcus pneumoniae*, and specific PCR for* Neisseria meningitidis*. All these PCR examinations were negative. Blood cultures from the time of admission showed no growth. On the second day of admission, the Laboratory of Clinical Microbiology at our hospital identified Gram positive rods from the CSF cultures. On suspicion of possible infection with* Listeria monocytogenes*, the antibiotical therapy was supplemented with intravenous gentamicin 240 mg daily. A CT of the brain with contrast and an MRI of the brain with contrast ruled out the presence of brain abscesses. During the next two days, she improved clinically with rising GCS. On the fourth day of admission, the Laboratory of Clinical Microbiology at our hospital confirmed identification of* Listeria monocytogenes* in the CSF. The* Listeria monocytogenes* strain was as expected sensitive to benzylpenicillin, ampicillin, and gentamicin. Treatment with ceftriaxone and aciclovir was terminated. She improved very fast and already on the fourth day she was up walking on the ward with a GCS of 15 points. The* Listeria monocytogenes* meningitis was treated for three weeks with high dose of benzylpenicillin, supplemented for the first week with gentamicin. The azathioprine treatment was halted during the admission, and after a few days of admission the oral prednisolone treatment was changed to intravenous hydrocortisone-succinat 100 mg two times daily. She had a flare-up in the level of her liver enzymes after two weeks of admission and she was switched back to oral prednisolone, starting out with a dose of 25 mg daily, but still pausing her azathioprine treatment. After three weeks of antibiotic treatment for* Listeria monocytogenes*, she was released from hospital. Seven months after the hospital stay, she was started again on her usual oral azathioprine 100 mg daily treatment combined with oral prednisolone 15 mg daily. A diagnostic work-up for immunodeficiencies was done and revealed a low IgG4 subclass 0.005–0.017 g/L (reference values 0.052–1.25 g/L). The IgG subclasses have been analyzed four times since her episode with* Listeria monocytogenes* meningitis (Table 1). Until now she has been clinically stable and she is followed up on a regular basis in both the Hepatology and the Infectious Diseases/Immune Defect Out-Patient Clinics at the National Hospital, Faroe Islands. She is still treated with azathioprine and prednisolone in the same dosages. She has not had any serious infections since her* Listeria monocytogenes* meningitis and she is living well.

## 3. Discussion

*Listeria monocytogenes* meningitis and/or sepsis is associated with the extreme of ages, immunosuppression, comorbidity, and pregnancy [1–7]. It is a very rare disease with an estimated incidence of 0.05–0.2 cases/100.000 population/yrs [8, 9]. The mortality rate is approx. 17–25% [1, 6, 10]. Ampicillin, amoxicillin, and benzylpenicillin are considered to be the treatment of choice for* Listeria monocytogenes* meningitis [1, 2, 6, 7]. A large recent multinational retrospective cohort study examined clinical features, diagnosis, treatment, and prognosis in neuroinfections with* Listeria monocytogenes* [6]. In this study, addition of aminoglycoside therapy did not affect the prognosis in neuroinfection with* Listeria monocytogenes* [6]. Delay in relevant antimicrobial treatment covering for* Listeria monocytogenes* and the presence of seizures were associated with increased mortality in this study [6].

Autoimmune hepatitis is an autoimmune disease involving the liver [11]. The disease is often well treated with prednisolone alone or combination treatment with azathioprine [11]. The incidence of autoimmune hepatitis has been reported to be 1.68/100.000 population/yrs in Denmark [12]. The patient in this case report was diagnosed with autoimmune hepatitis in 2006. At the time of admission with* Listeria monocytogenes* meningitis, she had been treated with prednisolone 5 mg daily and azathioprine 100 mg daily for several years, and she was clinically stable regarding her autoimmune hepatitis. To our best knowledge, no case has been published describing* Listeria monocytogenes* meningitis in a patient with autoimmune hepatitis and this is the first report with this combination of diseases. There is no convincing evidence in the literature associating autoimmune hepatitis with an increased risk of serious infections, independently of immunosuppressive drugs used to treat autoimmune hepatitis. However such a direct effect of the disease autoimmune hepatitis would be difficult to demonstrate, because most patients are treated with either prednisolone or prednisolone and azathioprine in combination. In other autoimmune diseases like systemic lupus erythematosus, there are data indicating an increased risk of infection independent of immunosuppressive treatment [13].

An increased risk of severe invasive infections and opportunistic infections is well documented in association with treatment with immunosuppressive drugs like prednisolone, azathioprine, tumour-necrosis-factor inhibitors (TNF-in), and other potent immunosuppressive drugs [14, 15]. TNF-in have been reported increasingly to be associated with different severe infections and opportunistic infections in patients treated with these potent immunosuppressive drugs [16]. More than 15 reports on cases of* Listeria monocytogenes* infection after treatment with TNF-in have been made [17]. In many of those cases, patients were also treated with other immunosuppressive drugs like prednisolone and azathioprine [17]. One report described occurrence of* Listeria monocytogenes* sepsis in a patient with ulcerative colitis treated with combination of prednisolone and azathioprine like in our patient [18]. Two reports described occurrence of* Listeria monocytogenes* in patients with ulcerative colitis treated with monotherapy of azathioprine [19–21]. Only one previous case with* Listeria monocytogenes* occurring in a patient with autoimmune hepatitis has been published [22]. This was spontaneous bacterial peritonitis with* Listeria monocytogenes* in a patient with liver cirrhosis, ascites, and autoimmune hepatitis treated with azathioprine [20, 21]. Our patient was treated with both prednisolone and azathioprine, so it is impossible to conclude which of those two immunosuppressive drugs had a main role in facilitating the development of* Listeria monocytogenes *meningitis in our patient. A synergistic negative role between prednisolone and azathioprine in our patient cannot be excluded. The role of her autoimmune hepatitis independently of the immunosuppressive drugs cannot be estimated. An immunological animal study on mice examining the negative effect on the immune system in coping infection with* Listeria monocytogenes*, when the immune system is exposed either to cyclophosphamide, vinblastine, and azathioprine or to methotrexate, indicated a substantial long-term negative effect of azathioprine on the immune response regarding infection with* Listeria monocytogenes* [23].

In a diagnostic and immunological work-up looking for other causes for this unusual infection in our patient with autoimmune hepatitis, we observed relative low levels of IgG4 subclass in screening of her immunoglobulin levels and her subclass IgG levels. A screening program for immune deficiencies in patients with neuroinfections has been done since 2011 at our department. All patients with life-threatening neuroinfections have been screened with measurements of immunoglobulins including IgG subclasses and flow cytometric analyses of lymphocyte subsets. That was the reason for measuring immunoglobulins in our patient presented in this report. The knowledge about the significance of low levels of IgG subclasses is still pending [24]. The most common IgG subclass deficiency is low levels of IgG2 combined with low levels of IgA [25]. IgG4 deficiencies are not common and the role of low levels of IgG4 has not been understood yet. Reports have been published showing that IgG4 deficiency can be a monodeficiency but can also occur with low levels of IgG2, IgA, and/or IgG1 [24, 25]. Deficiencies of IgG4 have been connected to different infections and autoimmune disorders; however data are scarce [26]. To our knowledge, this is the first report documenting presence of IgG4 deficiency and at the same time the combination of autoimmune hepatitis and* Listeria monocytogenes* meningitis. However, the role of this deficiency of IgG4 is pending. Several studies indicate that the treatment with prednisolone and azathioprine is probably not influencing the levels of IgG subclasses [27, 28]. This could indicate that the low IgG4 levels in our patient were independent of the immunosuppressive treatment with prednisolone and azathioprine, and therefore it could be speculated that our patient had an IgG4 deficiency immune defect that could have a role in the occurrence of* Listeria monocytogenes* meningitis. It is well known that levels of immunoglobulins including IgG subclasses can fluctuate significantly relating to active infection, operation, and other causes [25]. Therefore, measurements of IgG subclasses have to be repeated to come to a conclusion. The patient described in this report had also slightly elevated IgM and IgG3 subclasses and this can be explained by the autoimmune hepatitis where elevated levels of IgG and subclasses are seen [11].

In conclusion, we present the first case in the literature with the combination of* Listeria monocytogenes* meningitis and autoimmune hepatitis. Our report points towards three possible associations with the development of* Listeria monocytogenes* meningitis: her autoimmune hepatitis in itself; immunosuppressive treatment with prednisolone and azathioprine; and finally her IgG4 subclass deficiency.

## Figures and Tables

**Table 1:** Levels of immunoglobulins and subclasses.

Immunoglobulin reference values	January 2013	February 2013	September 2013	December 2014
IgG (total) g/L 6.4–13.5 g/L	9.8	11.0	12.3	12.1
IgA (total) g/L 0.7–3.12 g/L	1.95	2.63	2.90	2.58
IgM (total) g/L 0.56–3.52	2.80	4.22	5.11	3.73
IgG1 g/L 2.8–8 g/L	6.9	7.9	8.1	8.4
IgG2 g/L 1.2–5.7 g/L	2.18	2.52	2.92	2.89
IgG3 g/L 0.24–1.25 g/L	1.780	1.710	2.030	1.720
IgG4 g/L 0.052–1.25 g/L	0.006^a^	0.017^b^	0.005	0.015

^a^Sample from the third day of admission.

^b^Sample from 4 weeks after admission day.
